# Diabetes Mellitus 2014

**DOI:** 10.1155/2015/845759

**Published:** 2015-05-25

**Authors:** Ilias Migdalis, David Leslie, Anastasia Mavrogiannaki, Nikolaos Papanas, Paul Valensi, Helen Vlassara

**Affiliations:** ^1^2nd Medical Department and Diabetes Centre, NIMTS Hospital, 12 Monis Petraki, 11521 Athens, Greece; ^2^Department of Diabetes, Saint Bartholomew's Hospital, University of London and Blizard Institute, London EC1A 7BE, UK; ^3^Outpatient Clinic of the Diabetic Foot, Second Department of Internal Medicine, Democritus University of Thrace, 68100 Alexandroupolis, Greece; ^4^Department of Endocrinology, Diabetology and Nutrition, Jean Verdier Hospital, AP-HP, Paris Nord University, CRNH-IdF, CINFO, 93140 Bondy, France; ^5^Division of Experimental Diabetes and Aging, Mount Sinai School of Medicine, 1 Gustave L, Levy Place, P.O. Box 1460, New York City, NY 10029, USA

The ongoing progress in diabetes mellitus is reflected in new treatment algorithms [1], new oral hypoglycaemic agents [2], new tests for the early diagnosis of complications [3], and improved organisation of healthcare resources [4]. The present special issue is devoted to the recent progress. The articles cover 4 thematic areas: the main area is diabetic complications, while the other 3 are treatment, metabolism, and miscellaneous issues.

*(a) Diabetic Complications.* Complications of diabetes remain a major area needing improvement [4, 5]. J. Lowe et al. in their paper entitled “The Guyana Diabetes and Foot Care Project: Improved Diabetic Foot Evaluation Reduces Amputation Rates by Two-Thirds in a Lower Middle Income Country” reported an overall 68% reduction in major amputations after initiation of a multi-expert intervention programme in Guyana: below-knee amputations were reduced by 80%, while above-knee amputations remained unchanged. These results are very encouraging.

Two papers “Vascular Effects of Dietary Advanced Glycation End Products” by A. Stirban and D. Tschöpe and “Diabetes, Endothelial Dysfunction, and Vascular Repair: What Should a Diabetologist Keep His Eye on?” by V. Altabas shed more light on diabetes-induced vascular injury and the underlying mechanisms, including the effect of advanced glycation end products (AGEs) [6], depletion of nitric oxide (NO), and endothelial apoptosis.

Diabetic kidney function and diabetic nephropathy have been the object of some articles in this special issue. In their work titled “Inverse Levels of Adiponectin in Type 1 and Type 2 Diabetes Are in Accordance with the State of Albuminuria,” S. Ljubic et al. found that adiponectin increased in type 1 (T1DM) but decreased in type 2 diabetes (T2DM) with deterioration of albuminuria. These interesting results enrich our knowledge on the role of adiponectin as a risk marker of vascular disease in diabetes [7], while the difference between the two diabetes types calls for additional investigation. In 19 patients with diabetic nephropathy receiving angiotensin converting enzyme inhibitors or angiotensin II receptor blockers, S. M. Lee et al. evaluated the effect of omega-3 fatty acids (3 g per day) versus olive oil on proteinuria in their study entitled “Effect of Omega-3 Fatty Acid on the Fatty Acid Content of the Erythrocyte Membrane and Proteinuria in Patients with Diabetic Nephropathy.” Neither treatment succeeded in reducing proteinuria, possibly due to concomitant angiotensin converting enzyme inhibitor/angiotensin II receptor blocker treatment and to the absence of very severe proteinuria at baseline.

Including 150 patients with mild-to-moderate diabetic nephropathy, A. P. Silva et al. in their paper entitled “Low Magnesium Levels and FGF-23 Dysregulation Predict Mitral Valve Calcification as well as Intima Media Thickness in Predialysis Diabetic Patients” found that reduced magnesium and high levels of fibroblast growth factor-23 were independent predictors of mitral valve calcification and of increased carotid intima media thickness. These findings are a valuable contribution to the search for risk markers of vascular calcification in chronic kidney disease [8]. “The Blocking on the Cathepsin B and Fibronectin Accumulation in Kidney Glomeruli of Diabetic Rats” is a study by A. Wyczalkowska-Tomasik et al., who compared enalapril, losartan, enalapril plus losartan, spironolactone, and no treatment in terms of the activity of cathepsin B accumulation in the glomeruli. Enalapril plus losartan treatment increased glomerular activity of cathepsin B compared to untreated diabetic rats. Spironolactone increased this activity as well. These results need to be seen in the context of renin-angiotensin-aldosterone system inhibition as a therapeutic strategy for diabetic nephropathy, but caution is needed when extrapolating to humans.

Other groups dealt with coronary artery disease (CAD) and cardiac structure. K. Lalić et al. in their study entitled “Altered Daytime Fluctuation Pattern of Plasminogen Activator Inhibitor 1 in Type 2 Diabetes Patients with Coronary Artery Disease: A Strong Association with Persistently Elevated Plasma Insulin, Increased Insulin Resistance, and Abdominal Obesity” reported reduced diurnal fluctuation of plasminogen activator inhibitor-1 in patients with T2DM and CAD, along with increased insulin resistance, suggesting that these perturbations may be of relevance for the accelerated atherosclerosis in such patients. In their paper titled “The Difference Quantity of Urinary Peptides between Two Groups of Type 2 Diabetic Patients with or without Coronary Artery Disease,” G. Fu et al. examined T2DM patients and noted significant differences in the expression of peptides (fragments of isoform 1 of fibrinogen alpha chain precursor, prothrombin precursor, and inter-alpha-trypsin inhibitor heavy chain H4) between those with CAD and those without CAD, suggesting that we need to consider how such differences may be practically utilised in the search for CAD biomarkers. Similarly, E. C. Pereira et al. in their article entitled “Predictive Potential of Twenty-Two Biochemical Biomarkers for Coronary Artery Disease in Type 2 Diabetes Mellitus” found that 8 biomarkers (methionine, nitrate plus nitrite, n-acetyl-*β*-glucosaminidase, BMI, LDL, HDL, reduced glutathione, and L-arginine/asymmetric dimethyl-L-arginine) were associated with CAD in T2DM. Finally, K. Ziros et al. in their experimental study entitled “The effect of a low glycemic index diet on cardiac structure in an experimental model of diabetic rats” examined streptozotocin-induced diabetic rats and found greater area, perimeter and length of collagen fibres in heart vessels among those under normal glycaemic index diet as compared with those under low glycaemic index diet, indicating the importance of the glycaemic index of experimental diets in the study of complications in experimental works.

S. T.-H. Chiang et al. in their study entitled “Investigation of the Protective Effects of Taurine against Alloxan-Induced Diabetic Retinal Changes via Electroretinogram and Retinal Histology with New Zealand White Rabbits” demonstrated that taurine supplement reduced both hyperglycaemia and retinal electrophysiological changes in alloxan-induced diabetic rabbits. In a more practical setting, A. Jotic et al. in their article entitled “Decreased Insulin Sensitivity and Impaired Fibrinolytic Activity in Type 2 Diabetes Patients and Nondiabetics with Ischemic Stroke” identified the levels of insulin, plasminogen activator inhibitor-1, and insulin sensitivity as independent predictors of ischaemic stroke in patients with and without T2DM. The final study about complications is entitled “Role of the Insulin-Like Growth Factor Type 1 Receptor in the Pathogenesis of Diabetic Encephalopathy” by D. Zhang et al. The authors researched into diabetic encephalopathy, a condition including perturbations in cognition, cerebral signal conduction, neurotransmission, and other functions, in primary rat PC-12 cells. The authors found lower glucose metabolism and abnormally high expression of insulin-like growth factor-1 receptor in the diabetic encephalopathy model. The defect of insulin-like growth factor-1 receptor improved glucose utilisation and insulin sensitivity. These novel experimental results add to the accumulating experience on the role of insulin and insulin-like growth factor-1 in cognition and memory [9].

*(b) Treatment. *G. Rombopoulos et al. in their 24-week observational study of 659 metformin-treated T2DM patients titled “Treatment Compliance with Fixed-Dose Combination of Vildagliptin/Metformin in Patients with Type 2 Diabetes Mellitus Inadequately Controlled with Metformin Monotherapy: A 24-Week Observational Study” showed equal hypoglycaemic effect but higher compliance rates for the fixed vildagliptin plus metformin combination tablet versus the free-dose combination therapy with these two agents. I. Migdalis et al. performed a cost analysis of the treatment for T2DM in Greece (“The Cost of Managing Type 2 Diabetes Mellitus in Greece: A Retrospective Analysis of 10-Year Patient Level Data ‘The HERCULES Study'”). Of note, the largest part of total expenditure (48%) was for management of comorbidities, while pharmaceutical treatment comprised 35.9% and antidiabetic treatment only 14.9%. The highest cost was seen in obese men with a long diabetes duration and those with poor education. These useful results have obvious implications in the face of Greek economic crisis and its impact on the treatment of diabetic complications [10].

In their article entitled “Sitagliptin: Is It Effective in Routine Clinical Practice?” R. M. Dallumal et al. collected data from medical records of 457 T2DM patients. In the majority of these, sitagliptin was added to other antidiabetic agents, commonly metformin and/or sulfonylurea. The authors documented a −0.8% reduction in glycated haemoglobin within the first 6 months of sitagliptin treatment. However, no further improvement was seen during the next 6 treatment months. These observations are useful, but inhibitors of dipeptidyl peptidase 4 (DPP-4) usually maintain their efficacy longer [1], and so the authors need to look at longer follow-up data. H. Z. Huri et al. in their study entitled “Factors Associated with Utilization of Dipeptidyl-4 Inhibitors in Patients with Type 2 Diabetes Mellitus: A Cross-Sectional Retrospective Study” looked at retrospective data from 299 subjects taking either sitagliptin or vildagliptin. Of these, 95% received combination therapy. Age < 65 years (*p* = 0.049), no beta-blocker therapy (*p* = 0.045), and no aspirin therapy (*p* = 0.008) were significantly associated with choice of DPP-4 inhibitors as antidiabetic therapy. These observations reflect prescription patterns in the authors' country.

A. M. L. Martín et al. in their paper titled “Breaking Therapeutic Inertia in Type 2 Diabetes: Active Detection of In-Patient Cases Allows Improvement of Metabolic Control at Midterm” provided very interesting evidence that hospitalisation on the surgical ward may serve as an opportunity for detection of poor glycaemic control and prompt consultation with a diabetologist. Approximately half of the patients were reevaluated at 3–6 months, and a significant (*p* < 0.004) reduction in glycated haemoglobin was seen (mean reduction: −1.1%).

H.-W. Lin and C.-H. Tseng published “A Review on the Relationship between SGLT2 Inhibitors and Cancer.” Their conclusion was that the relationship between inhibitors of sodium-glucose cotransporter 2 and cancer is not conclusive, calling for larger databases and longer patient follow-up.

*(c) Metabolism.* This section covers various areas of metabolism. M. Acevedo et al. in their study entitled “Comparison of Lipoprotein-Associated Phospholipase A2 and High Sensitive C-Reactive Protein as Determinants of Metabolic Syndrome in Subjects without Coronary Heart Disease: In Search of the Best Predictor” observed that high sensitivity C-reactive protein (hsCRP) and Lipoprotein-associated phospholipase A2 (Lp-PLA2) were predictors of the metabolic syndrome in patients without CAD.

K. Toulis et al. in their study titled “Thyroid Autoimmunity in the Context of Type 2 Diabetes Mellitus: Implications for Vitamin D” examined an elderly population with frequent vitamin D deficiency and showed that the presence of T2DM increased the likelihood of thyroid autoimmunity. J. Tian et al. in their article entitled “Trends in the Levels of Serum Lipids and Lipoproteins and the Prevalence of Dyslipidemia in Adults with Newly Diagnosed Type 2 Diabetes in the Southwest Chinese Han Population during 2003–2012” demonstrated alarmingly high rates of dyslipidaemia, CAD, and cerebrovascular disease in newly diagnosed T2DM Chinese patients, emphasising the need for early diagnosis and aggressive management.

In their study entitled “Effects of Aerobic Exercise Based upon Heart Rate at Aerobic Threshold in Obese Elderly Subjects with Type 2 Diabetes,” G. P. Emerenziani et al. provided evidence that aerobic exercise improved physical fitness, heart function, and metabolism in obese elderly T2DM patients. A. S. Moghaddam et al. in their paper titled “The Effects of Soy Bean Flour Enriched Bread Intake on Anthropometric Indices and Blood Pressure in Type 2 Diabetic Women: A Crossover Randomized Controlled Clinical Trial” showed no significant effects of soy bread on anthropometric indices and blood pressure in T2DM. M. Rodríguez-Cruz et al. in their paper titled “Evidence of Insulin Resistance and Other Metabolic Alterations in Boys with Duchenne or Becker Muscular Dystrophy” found high rates of obesity and insulin resistance in boys with these two muscular dystrophies independent of corticosteroid treatment, while insulin resistance appeared to have a genetic component as well. In the experimental work “Resistance to the Beneficial Metabolic Effects and Hepatic Antioxidant Defense Actions of Fibroblast Growth Factor 21 Treatment in Growth Hormone-Overexpressing Transgenic Mice,” R. K. Boparai et al. noted resistance to the beneficial metabolic effects of fibroblast growth factor 21 in these transgenic animals.

*(d) Miscellaneous.* This final section covers diverse research fields. E.-H. Lee et al. published “Psychometric Properties of the Diabetes Management Self-Efficacy Scale in Korean Patients with Type 2 Diabetes,” providing a Korean version of the Diabetes Management Self-Efficacy Scale, which proved reliable for clinical use in their country. M. H. Yang et al. in their article “Do Behavioral Risk Factors for Prediabetes and Insulin Resistance Differ across the Socioeconomic Gradient? Results from a Community-Based Epidemiologic Survey” reported that the association between waist circumference and insulin resistance was independent of socioeconomic status, but its association with prediabetes was only significant in the highest socioeconomic level. These observations are interesting in terms of the interplay between societal and metabolic factors [11] and need further exploration. R. de M. B. Marques et al. in their article “Relative Validity and Reproducibility of a Quantitative Food Frequency Questionnaire for Adolescents with Type 1 Diabetes: Validity of a Food Frequency Questionnaire” showed adequate validity and reproducibility of a quantitative food frequency questionnaire for T1DM adolescents. A. Spirkova et al. in their work titled “Treated Autoimmune Thyroid Disease Is Associated with a Decreased Quality of Life among Young Persons with Type 1 Diabetes” found that thyroxin-treated autoimmune thyroid disease (but not coeliac disease) was associated with reduced quality of life in children with T1DM. These findings increase our insight into the intricate psychological issues needing careful consideration in the modern management of T1DM [12].

In the Norwegian study “The Chromosome 9p21 CVD- and T2D-Associated Regions in a Norwegian Population (The HUNT2 Survey),” Ø. Helgeland et al. confirmed the association of variants of* CDKN2B *on chromosome 9p21 in patients with T2DM and CAD. F. A. F. Da-Mata et al. in their paper “Prevalence of Self-Reported Diabetes and Its Associated Factors: A Population-Based Study in Brazil” reported a 10.1% prevalence of diabetes in the adult Brazil population. Age 35–65 years, hypertension, respiratory and cardiovascular disease, and pain/discomfort were significantly associated with diabetes. P. Mesquita et al. found a surprisingly high (20.6%) frequency of orthostatic hypertension in elderly patients with T2DM in their article entitled “Prevalence of Orthostatic Hypertension in Elderly Patients with Type 2 Diabetes.” Last but not least, A. S. Peacock et al. in their paper entitled “A Randomised Controlled Trial to Delay or Prevent Type 2 Diabetes after Gestational Diabetes: Walking for Exercise and Nutrition to Prevent Diabetes for You” documented that the use of a pedometer and a nutrition programme was successful in achieving both weight loss and increased physical activity over a 3-month period in women with prior gestational diabetes mellitus, cherishing the hope that this approach might contribute to the prevention of T2DM in later life.

*Conclusions*. This special issue testifies to the ongoing progress in diabetes research and care. Naturally, it has been impossible to cover all areas showing progress, for example, the role of microcirculation [13] and bariatric surgery [14]. More importantly, the challenge remains how much of and how often this new knowledge can be utilised in everyday clinical practice.

*Ilias Migdalis* *Ilias Migdalis* *David Leslie* *David Leslie* *Anastasia Mavrogiannaki* *Anastasia Mavrogiannaki* *Nikolaos Papanas* *Nikolaos Papanas* *Paul Valensi* *Paul Valensi* *Helen Vlassara* *Helen Vlassara*
